# Erythema Nodosum - An atypical presentation of melioidosis

**DOI:** 10.1590/0037-8682-0036-2022

**Published:** 2022-04-08

**Authors:** Peh Yi Tan, Jin Yi Goh

**Affiliations:** 1 Hospital Sultanah Nora Ismail, Department of General Medicine, Johor, Malaysia.

**FIGURE 1:**
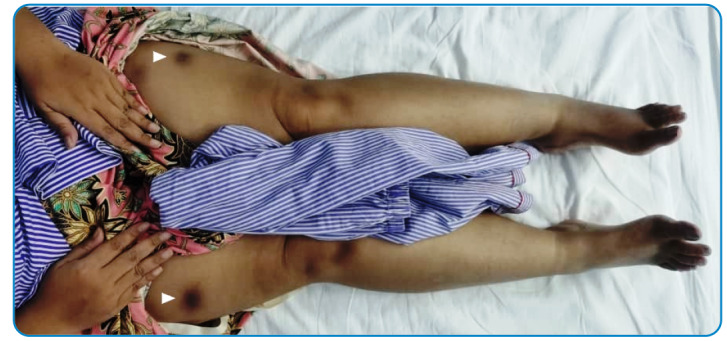
Erythema nodosum were seen on the bilateral thighs (white arrow).

**FIGURE 2:**
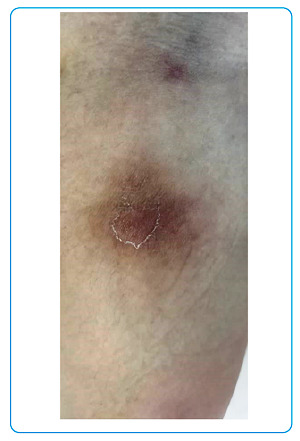
Image of a painful hyperpigmented skin nodule over the right forearm.

**FIGURE 3:**
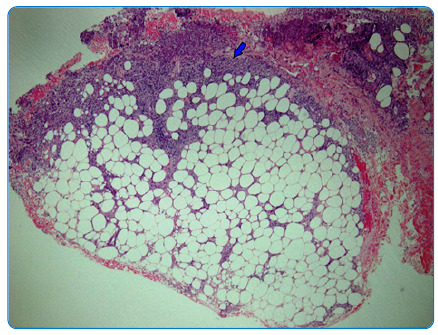
Histopathological examination of the skin biopsy specimen revealing dense septa panniculitis composed mainly of lymphohistiocytic infiltrates (blue arrow). 40X magnification.

A 39-year-old woman presented with fever for one month. Tender and erythematous subcutaneous nodules ranging from 3 to 6 cm in size were observed over the bilateral upper forearms, anterior thighs, and lower back region (Figure 1 and 2). The patient was diagnosed with type II diabetes mellitus during admission and as evidenced by 13.8% glycated hemoglobin. Blood cultures yielded *Burkholderia pseudomallei*, and melioidosis was diagnosed. A skin biopsy of the subcutaneous nodules showed dense septal panniculitis without any visible vasculitis or malignancy (Figure 3). No acid-fast bacilli or fungal bodies were detected using Ziehl-Neelsen or periodic acid-Schiff staining, respectively. Histological findings of the subcutaneous nodules were consistent with erythema nodosum (EN). She received intravenous ceftazidime for four weeks, followed by six months of trimethoprim-sulfamethoxazole. The EN resolved spontaneously after two months. 

EN is an uncommon and under-reported cutaneous manifestation of melioidosis^1^ ^-^ ^2^. It is a delayed hypersensitivity reaction that is triggered by various etiological factors, including tuberculosis, bacterial or deep fungal infection, sarcoidosis, leprosy, drugs, pregnancy, inflammatory bowel disease, or cancer. Classically, it is located in the bilateral pretibial area, but can also spread to the thighs, arms, and neck and occurs 3-5 times more often in women. The histological picture of EN shows septal panniculitis without vasculitis^3^. EN treatment is directed at the causative condition. Lesions typically resolve within 2-8 weeks without scarring. 

Recognizing EN as an atypical presentation of melioidosis is essential to prevent any delay in diagnosis and reduce morbidity.
